# Supplementary material for: Differential Tree Growth Response to Management History and Climate in Multi-Aged Stands of Pinus pinea L
Source: Plants (Basel). 2023 Dec 23;13(1):61. doi: 10.3390/plants13010061 (PMC10780625; doi:10.3390/plants13010061)
Supplement: Supplementary file 1 [file plants-13-00061-s001.zip › plants-2770976-supplementary.pdf]

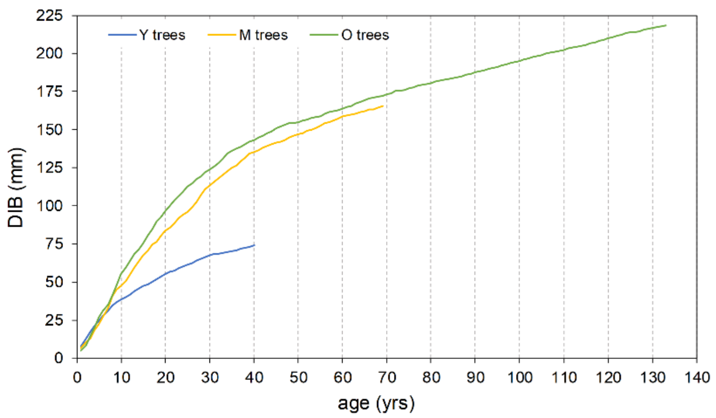

Figure S1. Cumulative DBI increment vs age of old (O), mature (M) and young (Y) trees obtained by averaging the individual series of each age class aligned at the same cambial age after estimating the number of missing rings from the pith.
